# Highly Cited Dental Implant Research: A Bibliometric Analysis of Publication Patterns and Research Themes

**DOI:** 10.3390/healthcare14101301

**Published:** 2026-05-11

**Authors:** Yuh-Shan Ho, Anastasios Grigoriadis, Nikolaos Christidis, Joannis Grigoriadis

**Affiliations:** 1 CT HO Trend, 3F.-7, No. 1, Fuxing N. Rd., Songshan Dist., Taipei City 105611, Taiwan; dr_ysho@hotmail.com; 2 Division of Oral Rehabilitation, Department of Dental Medicine, Karolinska Institutet, SE-14104 Huddinge, Sweden; nikolaos.christidis@ki.se (N.C.); joannis.grigoriadis@ki.se (J.G.)

**Keywords:** dental implants, bibliometric analysis, citation analysis, osseointegration, peri-implant diseases, clinical outcomes, research trends

## Abstract

**Highlights:**

**What are the main findings?**

- A total of 1599 highly cited dental implant publications were identified; after application of the ‘front page’ filter (n = 1324), 1016 original research articles were included, showing steady growth over time.
- Research is dominated by the United States and European institutions, with a strong emphasis on osseointegration and clinical outcomes.

**What are the implications of the main findings?**

- Citation patterns highlight key research leaders and influential research themes within implant dentistry.
- Bibliometric insights provide a descriptive overview of research activity and thematic development within implant dentistry.

**Abstract:**

**Background/Objectives:** Dental implant research has expanded substantially over recent decades, yet the characteristics of its most influential publications remain insufficiently defined. This study aimed to analyze highly cited dental implant research to identify global publication patterns, leading contributors, and major research themes. **Methods:** Publications indexed in the Web of Science Core Collection (SCI-EXPANDED) up to 1 May 2025 were retrieved. The analysis was restricted to WoSCC and used a fixed citation threshold; citation normalization and network analyses were not performed. Highly cited publications were defined as those with *TC*_2024_ ≥ 100 citations. A total of 1599 highly cited publications were identified; after application of the “front page” filter, 1324 implant-related documents remained, and 1016 original research articles were included in detailed bibliometric analyses. Citation indicators (*TC*_2024_, *CPP*_2024_) and authorship metrics (*FP*, *RP*, *IP*, *CP*, *SP*, and *APP*) were evaluated. Temporal trends, keyword frequency, and *Y*-index analysis were applied to assess research development and author contributions. **Results:** Publication output demonstrated steady growth, with peak citation impact observed after approximately 16 years. Research was primarily concentrated within dentistry, oral surgery, and biomedical-related fields. The United States and several European countries accounted for the majority of highly cited publications, with internationally collaborative articles showing higher citation impact than single-country publications. Keyword analysis identified four major research themes: biological integration, implant materials and surface characteristics, clinical treatment strategies, and peri-implant diseases. **Conclusions:** Highly cited dental implant research is characterized by strong representation of biological, material, and clinical domains. These findings should be interpreted as WoSCC-based citation patterns rather than definitive evidence of clinical impact, methodological quality, or global research priorities.

## 1. Introduction

Dental implant therapy is a well-established method for oral rehabilitation, yet long-term success depends on interactions between implant materials, biological responses, biomechanics, and clinical protocols. The concept of osseointegration provides the biological basis for implant stability and remains central to contemporary implant research [1,2,3]. Beyond biological integration, implant surface characteristics, biomechanical loading, and peri-implant tissue conditions also play critical roles in long-term outcomes [4,5,6,7,8,9]. Together, these factors have generated a broad and clinically important research field spanning biomaterials, surgery, prosthodontics, and complication management.

In bibliometric research, highly cited publications are commonly used to identify influential studies, major knowledge structures, and internationally visible research contributions [10,11,12,13,14]. In clinical fields, they also reflect evidence that has shaped treatment concepts, consensus development, and research priorities over time. However, citation counts are imperfect indicators of scientific influence. They may be affected by publication age, journal visibility, language, database coverage, research trends, collaboration networks, and self-citation. Therefore, highly cited publications should be interpreted as markers of citation visibility rather than direct measures of methodological quality, clinical importance, or research priority [15]. Accordingly, the present study is designed as a descriptive bibliometric analysis aimed at characterizing patterns of citation visibility rather than inferring research quality or priority.

However, citation data in the Web of Science Core Collection (WoSCC) are continuously updated, which can complicate reproducibility in bibliometric studies. To improve consistency, fixed citation indicators such as *TC_year_* have been proposed, allowing citations to be analyzed up to a defined cutoff year rather than using dynamic real-time counts [16].

Previous bibliometric studies in dentistry and related biomedical fields have mainly described general publication growth, citation performance, or selected subtopics, whereas the highly cited segment of dental implant research has not been systematically characterized with respect to influential publications, leading contributors, and dominant research priorities [17,18,19,20]. This limits understanding of which studies, authors, and research areas have achieved the greatest citation visibility within implant dentistry. Therefore, this study aimed to analyze highly cited dental implant publications to characterize global research trends, identify highly cited contributors, and describe major research themes within the literature.

The remainder of this article is structured as follows. Section 2 describes the study design, data source, search strategy, eligibility criteria, and bibliometric indicators. Section 3 presents the main findings, including publication characteristics, temporal trends, leading contributors, and thematic patterns. Section 4 summarizes the main conclusions.

## 2. Materials and Methods

### 2.1. Study Design and Reporting Guideline

This bibliometric study was designed and reported in accordance with the BIBLIO checklist (Table S1), which provides minimum reporting requirements for bibliometric reviews of the biomedical literature [21]. The study design followed established bibliometric approaches to ensure transparency and reproducibility.

### 2.2. Data Source and Search Strategy

Data were obtained from the Web of Science Core Collection (SCI-EXPANDED; Clarivate Analytics), with the latest update on 1 May 2025. All data were retrieved on the same date to ensure consistency of citation counts. The literature search covered publications from 1991 to 2024 and was performed using a Topic (TS) search with Boolean operators to retrieve records containing at least one predefined implant-related term. The final search string used in the Web of Science Core Collection was TS = (“dental implant” OR “dental implants” OR “implant dentistry” OR “oral implantology” OR “dental implantology” OR “implant prosthodontics” OR “dental implant surgery” OR “dental implant surgeries”). The Topic (TS) field includes title, abstract, author keywords, and *Keywords Plus*.

WoSCC was selected because it provides structured citation data, standardized journal indexing, and compatibility with fixed citation indicators, such as *TC*_2024_. Nevertheless, reliance on a single database may introduce selection bias, and the results should not be generalized to all dental implant literature indexed in Scopus, PubMed, or Google Scholar.

### 2.3. Eligibility Criteria and Identification of Highly Cited Publications

Highly cited publications were defined as documents that had received at least 100 citations in the WoSCC from the year of publication through 2024 (*TC*_2024_ ≥ 100). This threshold was selected to focus on the most influential segment of the literature, as commonly applied in bibliometric studies to identify highly cited publications. The threshold of *TC*_2024_ ≥100 was used to define a highly cited subset and to allow comparison with previous bibliometric studies. This cutoff is pragmatic rather than field-normalized; therefore, the analysis does not claim to identify all influential publications or to rank research quality.

**Figure 1:**
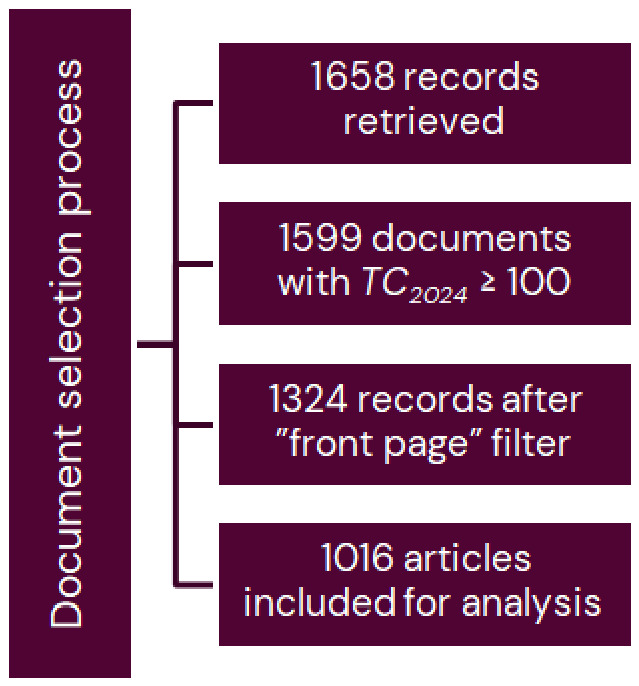
Flowchart of document selection process.

Following application of the search strategy, records containing the predefined keywords in the TOPIC field were retrieved from the SCI-EXPANDED database. For the period between 1991 and 2023, 1658 records were retrieved. Among these, 1599 documents (96%) with *TC*_2024_ ≥ 100 citations were identified as highly cited publications. Following application of the ‘front page’ filter, 1324 highly cited implant-related documents remained. For detailed bibliometric analyses, only original research articles were included, resulting in a final dataset of 1016 articles, as shown in Figure 1. Complete records, including annual citation data, were exported to Microsoft 365 Excel (Redmond, WA, USA) for subsequent analysis [22]. No independent full-text manual screening was performed. Relevance was improved by applying the ‘front page’ filter, which restricts records to those containing the search terms in the title, abstract, or author keywords. However, this approach cannot fully eliminate false positives or recover false negatives, and this limitation is acknowledged [23].

### 2.4. Data Extraction and Bibliometric Indicators

Publication performance was assessed using citation indicators including *C*_year_, *TC*_year_, and *CPP*_year_. The indicator *C*_year_ represents the number of citations received in a specific year, for example, *C*_2024_ reflects citations recorded in 2024 [23]. *TC*_2024_ refers to the cumulative number of citations from the year of publication up to the end of the most recent year included in the analysis, which in this study corresponds to *TC*_2024_. *CPP*_year_ represents the average citation impact per publication and was calculated as *TC*_2024_ divided by the total number of publications (*TP*) [24]. In addition, to further characterize the dataset, the complementary indicator *APP* (average number of authors per publication) was used to describe document characteristics and collaboration patterns in highly cited publications [25]. These indicators were applied to analyze publication output, citation impact, and distributions across journals, countries, institutions, and articles. Finally, the analysis of word frequency in article titles and author keywords has been proposed as an effective approach for identifying main research themes [26].

### 2.5. Authorship Attribution and Affiliation Standardization

Authorship roles were classified based on WoSCC conventions, where the reprint author corresponds to the corresponding author; however, the term “corresponding author” was used consistently throughout the study. The first and corresponding authors are generally recognized as the principal contributors to a scholarly publication, with their roles often indicating substantial intellectual input and responsibility [27]. In single-author publications involving one institution and one country, where explicit authorship roles were not specified, the author was treated as both first and corresponding author, and the associated institutional and national affiliations were assigned accordingly. When more than one corresponding author was listed, all relevant authors, together with their institutional and national affiliations, were included in the analysis. To improve data consistency, affiliation information was carefully reviewed and standardized [23]. Furthermore, geographical classifications were unified, with regions such as England, Scotland, Northern Ireland, and Wales categorized under the United Kingdom.

### 2.6. Temporal Analysis of Publication Output

Temporal changes in publication output and citation impact were analyzed by examining the relationship between annual publication counts (*TP*) and citations per publication (*CPP_year_*) [28]. This approach made it possible to identify patterns over time in both research productivity and citation impact.

### 2.7. Country-, Institution-, and Author-Level Indicators

Publication performance at the country and institutional levels was evaluated using six commonly applied indicators [29,30], including total output (*TP*), independent publications (*IP*), collaborative publications (*CP*), first-author publications (*FP*), corresponding-author publications (*RP*), and single-author publications (*SP*). Independent and collaborative publications were defined at both the country level (*IP_C_* and *CP_C_*) and institutional level (*IP_I_* and *CP_I_*).

To further assess research performance across countries, these publication indicators were analyzed together with citation impact (*CPP*_2024_) across different document types, journals, and subject categories [31]. It should be noted that journals in the WoSCC may be assigned to more than one subject category. For example, *Materials* is classified under physical chemistry, multidisciplinary materials science, metallurgy and metallurgical engineering, applied physics, and condensed matter physics. Category-based analyses were therefore conducted with the understanding that cumulative percentages may exceed 100% [22]. In addition, journal characteristics, such as citations per publication (*CPP_year_*) and authors per publication (*APP*), are widely used indicators for describing journal performance in bibliometric studies [32].

### 2.8. Y-Index Analysis

The *Y*-index (*j*, *h*) was used to evaluate author contributions, where j represents the total number of first- and corresponding-author publications, and h reflects the distribution between these roles [23,33].

### 2.9. Generative Artificial Intelligence in Manuscript Preparation

No generative artificial intelligence (GenAI) tools were used in the preparation of this manuscript.

## 3. Results

### 3.1. Document Type and Language of Publication

A total of 1324 highly cited dental implant documents in the SCI-EXPANDED database, each with *TC*_2024_ values of 100 or more citations, were identified. Table 1 presents the characteristics of the six identified document types, including the total number of publications (*TP*), *APP*, and *CPP*_2024_. Because Web of Science document types are not mutually exclusive, the sum of document types (1414) exceeds the total number of publications (1324). Among these, articles were the most prevalent document type, comprising 1016 records (77% of the 1324 highly cited documents), with an *APP* of 5.0 authors per publication. Highly cited reviews, while fewer in number, demonstrated the highest impact with a *CPP*_2024_ of 227 citations per publication, slightly exceeding that of articles, which had a *CPP*_2024_ of 189 citations. This indicates that review articles may have a higher citation impact within the dataset. These findings provide insight into the structure and citation impact of influential research within implant dentistry.

A total of 302 highly cited reviews were published in a wide range of 98 journals. The majority appeared in *Clinical Oral Implants Research* (*IF*_2023_ = 4.8) (27 reviews; 8.9% of 302 highly cited reviews), with a *CPP*_2024_ of 216 citations per publication, followed by the *International Journal of Oral & Maxillofacial Implants* (*IF*_2023_ = 1.7) (25 reviews; 8.3% of 302 highly cited reviews), with a *CPP*_2024_ of 209 citations and the *Journal of Periodontology* (*IF*_2023_ = 4.2) (23; 7.6%), with a *CPP*_2024_ of 216 citations. Four of the eleven classic publications with *TC*_2024_ of 1000 or more citations [34] in dental implant research were reviews by Le Guéhennec et al. (2007), Zhou and Lee (2011), Anitua et al. (2024), and Puleo and Nanci (1999) with *TC*_2024_ of 1879, 1239, 1085, and 1067 citations, respectively [35,36,37,38].

All 1016 highly cited articles related to dental implant research were published in English.

### 3.2. Publication Distribution

**Figure 2:**
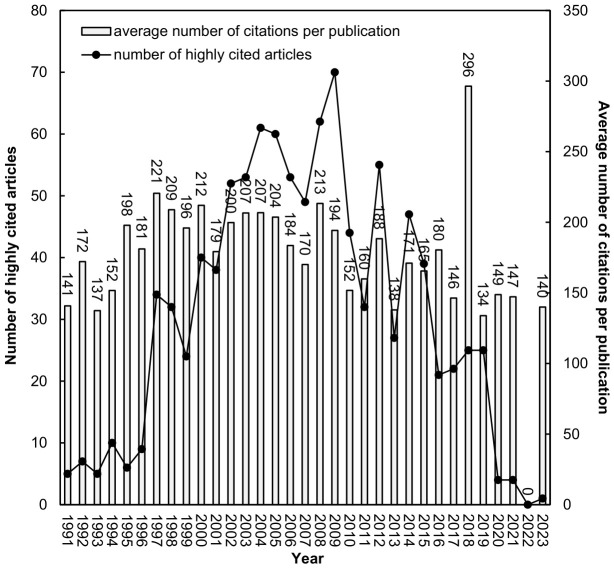
This figure provides a descriptive overview of temporal distribution and citations per publication by 2024 (*CPP*_2024_). No formal trend test was applied; therefore, apparent temporal changes should be interpreted cautiously.

In total, 1016 highly cited dental implant-related articles published between 1991 and 2023 were identified, with a *TC*_2024_ of 191,622 citations and a *CPP*_2024_ of 189 citations per publication. The highest *TC*_2024_ observed was 1407 citations for a single article. Figure 2 illustrates the annual distribution of these 1016 articles along with their corresponding *CPP*_2024_ values, showing temporal trends in publication output and citation impact within implant research. Only five highly cited articles were published in 1991. The latest highly cited article was “Prevention and treatment of peri-implant diseases: The EFP S3 level clinical practice guideline” [39] with a *TC*_2024_ of 140 citations. Herrera et al. (2023) [39] reported clinical practice guidelines addressing peri-implant disease management.

The annual distribution showed that the number of highly cited articles reached a peak of 70 publications after approximately 16 years. In 2018, 25 articles were published, with the highest CPP2024 (296 citations per publication). Several of the most highly cited publications that year addressed peri-implant disease classification and management [9,40,41].

### 3.3. Web of Science Categories and Journals in SCI-EXPANDED

A total of 127 journals in 56 Web of Science categories in SCI-EXPANDED published highly cited articles related to dental implants. The top three productive Web of Science categories—‘dentistry, oral surgery and medicine,’ ‘biomedical engineering,’ and ‘biomaterials science’—published 926 (91% of 1016) highly cited articles. Most articles were classified in dentistry, oral surgery, and biomedical-related categories. The category of ‘dentistry, oral surgery and medicine’ is the predominant category, housing 91 journals in SCI-EXPANDED in 2023 and accounting for 80% of the total articles (812 out of 1016 articles), followed by categories of ‘biomedical engineering’ and ‘biomaterials materials science’ with 389 (38%) and 119 (12%) highly cited articles.

Table 2 presents the top 10 most productive journals, each publishing 20 or more highly cited articles. Among these, eight journals belong to the category of ‘dentistry, oral surgery and medicine,’ three in ‘biomedical engineering,’ and two in ‘biomaterials materials science.’ Clinical Oral Implants Research (*IF*_2023_ = 4.8) published the highest number of highly cited articles, with 278 articles, accounting for 27% of the total 1016 highly cited articles.

The comparison of the top productive journals in Table 2 indicates that 64 articles published in the Journal of Clinical Periodontology (*IF*_2023_ = 5.8) achieved the highest *CPP*_2024_, with 252 citations per publication, while the 39 highly cited articles in the Clinical Implant Dentistry and Related Research (*IF*_2023_ = 3.7) had a *CPP*_2024_ of 157 citations per publication. The average number of authors per publication (*APP*) ranged from 6.4 authors per publication in Biomaterials to 4.0 authors in the International Journal of Oral & Maxillofacial Implants.

Based on the 2023 journal impact factors (*IF*_2023_), only two journals had *IF*_2023_ more than 15, namely Periodontology 2000 (*IF*_2023_ = 17.5) and Science Translational Medicine (*IF*_2023_ = 15.8), with three and two highly cited articles, respectively.

### 3.4. Countries, Institutions, and Authors

Within the SCI-EXPANDED database, 5 out of 1016 highly cited dental implant-related articles (0.49%) lacked author affiliation information. The remaining 1011 articles were authored by researchers affiliated with institutions in 53 different countries. Of these, 694 articles (69%) were classified as single-country articles originating from 36 countries, with a *CPP*_2024_ of 184 citations per article. The remaining 317 articles (31%) were internationally collaborative, involving co-authors from 50 countries, and had a higher citation impact, with a *CPP*_2024_ of 201 within this dataset. Internationally collaborative articles had a higher *CPP*_2024_ (201) compared to single-country articles (184).

Table 3 summarizes data for the 10 most productive countries, each with 50 or more highly cited articles. The list includes eight European countries and one each in the Americas and Asia, respectively. Australia was the most productive in Oceania, with 27 articles (ranked 14th). Egypt was the only country in Africa with two internationally collaborative, highly cited articles. Five of the top ten countries—the USA, Sweden, the UK, Switzerland, and Italy—had single-author articles.

The USA had the highest values across all six publication indicators:

- Total Publications (*TP*): 308 highly cited articles (30% of 1011 highly cited articles).
- Independent Publications (*IP*_C_): 152 articles (22% of 694 single-country highly cited articles).
- Collaborative Publications (*CP*_C_): 156 articles (49% of 317 internationally collaborative highly cited articles).
- First-Author Publications (*FP*): 219 articles (22% of 1011 first-author highly cited articles).
- Corresponding-Author Publications (*RP*): 216 articles (22% of 1004 corresponding-author highly cited articles).
- Single-Author Publications (*SP*): 10 articles (40% of 25 single-author highly cited articles).

Among the top 10 countries, Switzerland, with a *TP* of 196 articles, a *CP*_C_ of 124 articles, an *FP* of 122 articles, and an *RP* of 122 articles, had the greatest *CPP*_2024_ of 225, 235, 227, and 226 citations per publication for total highly cited articles, internationally collaborative articles, first-author articles, and corresponding-author articles, respectively. The UK, with an *IP*_C_ of 20 articles and an *SP* of 3 articles, had the greatest *CPP*_2024_ of 201 and 307 citations per publication for single-country articles and single-author articles, respectively. Furthermore, Italy and the Netherlands had lower citations for the highly cited dental implant-related articles.

Of the 1011 highly cited dental implant-related articles in the SCI-EXPANDED database, 382 articles (38%) were published by a single institution and had a *CPP*_2024_ of 187 citations per publication. The remaining 629 articles (62%) were the result of inter-institutional collaborations, which recorded a slightly higher *CPP*_2024_ of 190 citations.

Table 4 presents the publication characteristics of the 10 most productive institutions with at least 24 highly cited articles. Among them, four institutions were from the USA, two from Switzerland, two from Sweden, and one each from Italy and China. Only four of the top ten institutions had single-author articles.

The University of Bern (Switzerland) had the highest values in five of the six publication indicators:

- Total Publications (*TP*): 126 highly cited articles (12% of 1011 highly cited articles).
- Independent Publications (*IP*_I_): 28 articles (7.3% of 382 single-institution articles).
- Institutionally Collaborative Publications (*CP*_I_): 98 articles (16% of 629 inter-institutionally collaborative articles).
- First-Author Publications (*FP*): 67 articles (6.6% of 1011 first-author articles).
- Corresponding-Author Publications (*RP*): 63 articles (6.4% of 992 corresponding-author articles).

Furthermore, Aarhus University (Denmark) and Astra Tech AB (Sweden) were the only institutions that had two highly cited single-author articles.

Among the top 10 institutions, the University of Michigan (USA), with a *TP* of 34 articles and a *CP*_I_ of 24 articles, had the greatest *CPP*_2024_ of 263 and 307 citations per publication for total highly cited articles and inter-institutionally collaborative articles, respectively. The University of Bern (Switzerland), with an *IP*_I_ of 28 articles, had the greatest *CPP*_2024_ of 244 citations per publication for single-institution articles. The University of Zurich (Switzerland), with an *FP* of 26 articles, had the greatest *CPP*_2024_ of 229 citations per publication for first-author articles. The University of Gothenburg (Sweden), with an *RP* of 41 articles, had the greatest *CPP*_2024_ of 228 citations per publication for corresponding-author articles. The University of Milan (Italy), with an *SP* of one article, had the greatest *CPP*_2024_ of 123 citations per publication for single-author articles.

For 1016 highly cited dental implant-related articles, the *APP* was recorded at 5.0 authors per publication with a maximum of 42 authors in an article titled “Group 5 ITI Consensus Report: Digital technologies” published in 2018 [42]. Wismeijer et al. (2018) [42] authored the article, boasting 14 countries and 21 institutions. A total of 711 highly cited articles had corresponding author information in the SCI-EXPANDED database. Only 15 highly cited articles were published by multiple corresponding authors. Of the 1016 highly cited dental implant-related articles, 79% articles were published by groups of two to six authors, including 256 highly cited articles (25% of 1016 articles), 187 articles (18%), 140 articles (14%), 131 articles (13%), and 91 articles (9.0%) written by groups of 4, 5, 6, 3, and 2 authors, respectively.

Table 5 lists the top 20 most productive authors with three publication indicators, their citation indicators, and *Y*-index constants [43]. N.P. Lang was the most productive author, boasting an impressive 46 highly cited articles, among which two were authored as the first author and three as the corresponding author. R.E. Jung, M.M. Bornstein, T. Berglundh, and D. Buser published the most (nine) first-author articles, respectively. T. Berglundh published the most (15) corresponding-author articles. Compared to the top 20 authors in Table 5, F. Schwarz, with a *TP* of 16 highly cited articles, had the greatest *CPP*_2024_ of 295 citations per publication for total articles. D. Buser, with an *FP* of nine articles, had the greatest *CPP*_2024_ of 502 citations per publication for first-author articles. R.E. Jung, with an *RP* of 9 articles, had the greatest *CPP*_2024_ of 316 citations per publication for corresponding-author articles. Nine of the twenty productive authors, including D. Buser, T. Berglundh, R.E. Jung, D.L. Cochran, H.L. Wang, A. Piattelli, M. Quirynen, F. Schwarz, and M.M. Bornstein, were also found to be the top 20 publication potential authors in cochlear implants research as evaluated by *Y*-index.

A total of 711 (70% of 1016) highly cited dental implant-related articles in SCI-EXPANDED included both first- and corresponding-author information and were used to calculate the *Y*-index for individual authors. These articles were contributed to by 2439 unique authors. Among them, 1741 authors (71%) did not contribute as either first or corresponding author and were therefore assigned a *Y*-index of (0, 0). For example, J. Lindhe authored 14 highly cited articles but had a *Y*-index of (0, 0), indicating no first- and corresponding-author role. A total of 150 authors (6.2%) published only as corresponding authors, with *h* = π/2. One such example is X.Y. Liu, who had a *Y*-index of (5, π/2) based on five corresponding-author articles. A total of 37 authors (1.5%) published a greater number of corresponding-author articles than first-author articles (π/2 > *h* > π/4) with *FP* > 0. Meanwhile, 301 authors (12%) had equal numbers of first- and corresponding-author articles, resulting in *h* = π/4. Only 12 authors (0.49%) published more first-author articles than corresponding-author articles (π/4 > *h* > 0) with *RP* > 0. In contrast, 198 authors (8.1%) published only as first authors (*h* = 0).

**Figure 3:**
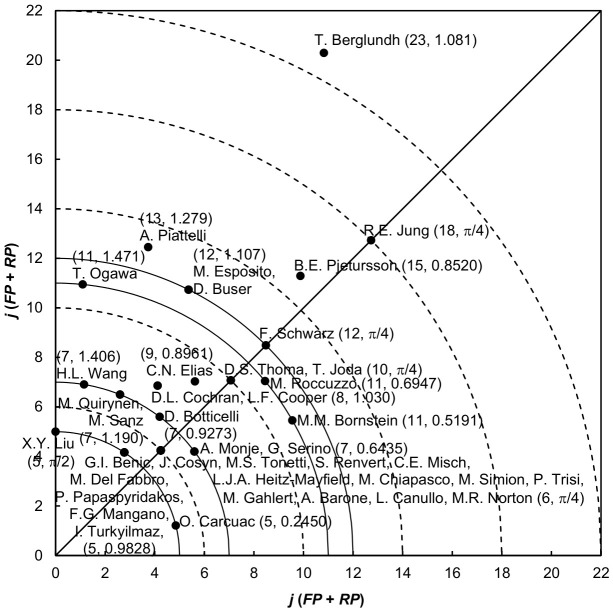
Because the *Y*-index is a specialized bibliometric indicator, its interpretation is limited to authorship-role patterns and should not be interpreted as a measure of overall scientific quality or clinical influence.

Figure 3 illustrates the distribution of *Y*-indices for the 40 highly cited authors with a *j* of 5 or more. Each point on the plot represents a unique *Y*-index value that may correspond to one or multiple authors. For instance, J. Cosyn, M.S. Tonetti, S. Renvert, C.E. Misch, L.J.A. Heitz-Mayfield, M. Chiapasco, M. Simion, P. Trisi, M. Gahlert, A. Barone, L. Canullo, and M.R. Norton shared the same *Y*-index of (6, π/4), reflecting identical publication potential and characteristics. T. Berglundh, with a *Y*-index of (23, 1.081), had the highest publication potential (*j* = 23), followed by R.E. Jung and B.E. Pjetursson with a *j* of 18 and 15, respectively.

Authors such as X.Y. Liu (5, π/2), G.I. Benic, four other authors (5, 0.9828), and O. Carcuac (5, 0.2450) were located on the same curve with the same *j* of 5 but different *h* values, indicating the same publication potential but different authorship roles. Liu published only corresponding-author articles with an *h* of π/2, Benic and four other authors published a higher ratio of corresponding-author articles to first-author articles with an *h* of 0.9828, and Carcuac published a higher ratio of first-author articles to corresponding-author articles with an *h* of 0.2450. Similarly, H.L. Wang (7, 1.406), M. Quirynen, M. Sanz (7, 1.190), D. Botticelli (7, 0.9273), and A. Monje, and G. Serino (7, 0.6435) all lie on the *j* = 7 curve, with Wang publishing the highest ratio of corresponding-author articles to first-author articles with an *h* of 1.406, followed by Quirynen and Sanz with an *h* of 1.190, and Botticelli with an *h* of 0.9273. However, Monje and Serino published a higher ratio of first-author articles to corresponding-author articles with an *h* of 0.6435.

Authors along the diagonal line (*h* = π/4), such as R.E. Jung (18, π/4), F. Schwarz (12, π/4), D.S. Thoma and T. Joda (10, π/4), and J. Cosyn and 11 other authors (6, π/4) all had equal contributions as first and corresponding authors but differed in overall publication potential (*j*)m following the order of Jung with a *j* of 18, Schwarz (*j* = 12), Thoma and Joda (*j* = 10), and Cosyn et al. (*j* = 6).

In summary, the distribution of authors in Figure 3 reflects distinct families of publication characteristics (as indicated by *h*) and publication potential (as indicated by *j*). These results provide additional insight into authorship roles and leadership patterns within highly influential research. Authors with the same *j* value lie on the same curve, while those with the same *h* value fall along the same angular direction from the origin.

Key contributions to dental implant research were made by Per-Ingvar Brånemark, who introduced osseointegration as the biological basis for titanium implants [2]. Tomas Albrektsson defined implant success criteria and bone–implant biology [3,44]. Ann Wennerberg clarified the role of surface topography [5], while Thomas Berglundh advanced peri-implant disease classification [9,45]. Internationally, Daniel Buser and Niklaus P. Lang made major contributions to implant surgery, bone regeneration, and peri-implant disease prevention [46,47], while Christoph H. F. Hämmerle advanced guided bone regeneration and implant treatment concepts [48].

### 3.5. Analysis of Words in Highly Cited Article Title and Author Keywords

Table 6 presents the 20 most frequently used words in the titles and author keywords of highly cited dental implant-related research, excluding the predefined search keywords. Based on the most frequently used words, four main themes emerged and are presented below.

#### 3.5.1. Theme 1: Biological Integration and Bone–Implant Interactions

Frequently occurring keywords in this theme included bone, osseointegration, bone regeneration, bone loss, histology, osteoblasts, tissue, and hydroxyapatite.

#### 3.5.2. Theme 2: Implant Materials and Surface Characteristics

Keywords associated with this theme included titanium, surface, implant stability, accuracy, and influence.

#### 3.5.3. Theme 3: Clinical Treatment Strategies and Surgical Techniques

Frequently occurring keywords in this theme included clinical, patients, treatment, placement, immediate loading, sinus, sinus floor elevation, augmentation, maxillary, and edentulous.

#### 3.5.4. Theme 4: Peri-Implant Diseases, Complications, and Evidence-Based Evaluation

Keywords associated with this theme included peri-implantitis, peri-implant, periodontitis, complications, biological complications, survival, systematic review, clinical trial, prospective, and trial.

## 4. Discussion

This bibliometric analysis provides a comprehensive overview of highly cited dental implant research, highlighting publication trends, influential contributors, and dominant research themes. The findings demonstrate that highly cited publications are concentrated within a limited number of journals, countries, institutions, and author groups, reflecting established centers of expertise and sustained research activity within implant dentistry.

The temporal distribution of highly cited publications showed that peak citation impact occurred approximately 16 years after publication. This pattern may be associated with the time required for clinical validation, long-term follow-up, and the integration of research findings into clinical practice. Unlike rapidly evolving biomedical fields, implant dentistry often relies on longitudinal studies and outcome-based evidence, which may contribute to delayed citation peaks [49,50]. A transient increase in citation impact was also observed for articles published around 2018, many of which focused on peri-implant disease classification and management [9,40,41]. This pattern may reflect the influence of consensus reports and clinically adopted disease frameworks on citation behavior.

These findings are broadly consistent with previous bibliometric studies in dentistry and related biomedical fields, which have also shown a concentration of influential research within a limited number of countries, journals, and research groups [17,18,19,20]. However, the present study extends this perspective by focusing specifically on the highly cited segment of dental implant research. While these patterns provide insight into the structure of highly cited implant research, they should be interpreted with caution. Citation distributions are shaped not only by scientific contribution but also by structural and behavioral factors within the scientific system. For example, publications from high-impact journals, large collaborative networks, and widely recognized research groups may achieve greater visibility and citation accumulation independent of methodological quality. Similarly, consensus reports, guidelines, and review articles tend to receive disproportionately high citation counts due to their broad applicability. In addition, self-citation and disciplinary citation practices may further influence citation patterns. Therefore, the observed concentration of highly cited publications within specific countries, institutions, and thematic areas likely reflects a combination of scientific contribution and systemic citation dynamics rather than purely research excellence.

The analysis of publication types revealed that review articles exhibited higher citation impact compared to original research articles. This may reflect the role of reviews in synthesizing existing knowledge and providing comprehensive overviews that are widely cited across different subfields. In addition, the presence of highly cited consensus reports and clinical guidelines suggests that publications with broad clinical applicability may receive greater attention within the scientific community.

Several of the most highly cited publications identified in this study focused on peri-implant disease classification and management, including the consensus report by Tord Berglundh and related studies [9,40,41]. These works addressed diagnostic criteria, disease classification, and treatment approaches, and may reflect the increasing recognition of peri-implant diseases as a major clinical challenge. This interpretation is consistent with epidemiological evidence showing that peri-implant diseases are common in treated populations [51]. Similarly, clinical practice guidelines, such as those reported by David Herrera et al. [39], have contributed to the standardization of peri-implant disease management and may influence citation patterns due to their broad clinical relevance.

At the country level, the results showed that the United States and several European countries accounted for the majority of highly cited publications. Internationally collaborative articles demonstrated higher citation impacts compared to single-country publications, which may be related to increased visibility, resource sharing, and broader research networks. A similar association between collaboration and research impact has been observed across multiple scientific fields [52]. Among European countries, Switzerland and Sweden contributed substantially to highly cited dental implant research, both in terms of publication output and citation impact. Switzerland, in particular, showed consistently high citation impact across multiple publication indicators, which may reflect strong research specialization within implant-related fields. At the institutional level, the University of Bern was one of the most productive contributors, demonstrating high values across several publication indicators. Other institutions, including the University of Gothenburg and the University of Zurich, also contributed prominently to highly cited research output. These findings indicate that highly cited implant research is concentrated within a limited number of established academic centers with sustained research activity [2,46,48,53,54]. This concentration of highly cited research within specific countries and institutions may reflect long-term investment in implant research infrastructure and established collaborative networks. Similarly, inter-institutional collaborations showed slightly higher citation impacts than single-institution studies, suggesting that collaborative research environments may be associated with increased dissemination and citation.

The analysis of journals and subject categories indicated that highly cited dental implant research is primarily concentrated within dentistry, oral surgery, and biomedical-related fields. This distribution is consistent with the interdisciplinary nature of implant dentistry, which integrates biological, material, and clinical sciences. The prominence of journals such as *Clinical Oral Implants Research* and the *Journal of Clinical Periodontology* further highlights the importance of specialized journals in disseminating influential research within this field.

The thematic analysis of keywords identified four major research areas: biological integration, implant materials and surface characteristics, clinical treatment strategies, and peri-implant diseases. The prominence of keywords related to osseointegration reflects the fundamental role of biological integration in implant success. In addition, the frequent occurrence of implant surface-related terms suggests sustained research interest in optimizing material properties to enhance clinical performance.

Keywords related to clinical outcomes, including survival rates and complications, indicate an ongoing focus on long-term treatment evaluation. Furthermore, the presence of peri-implant disease-related terms, such as peri-implantitis and mucositis, reflects increasing attention to biological complications and their management [45]. Together, these findings indicate that highly cited research in implant dentistry is concentrated on fundamental biological mechanisms, material optimization, and long-term clinical outcomes.

Authorship analysis using the *Y*-index provided additional insight into research leadership and contribution patterns. A relatively small group of authors contributed a substantial proportion of highly cited publications, reflecting concentrated expertise and sustained research productivity within the field. The distribution of first- and corresponding-author roles further highlights differences in leadership patterns and collaboration structures among highly productive researchers.

These findings should be interpreted within the context of citation-based analysis. Citation counts may be influenced by factors such as publication age, journal visibility, research trends, and collaboration networks, and do not necessarily reflect methodological quality or clinical effectiveness. In addition, the use of a citation threshold may favor older publications that have had more time to accumulate citations.

### Study Limitations and Strengths

This study has several limitations. The analysis was limited to the Web of Science Core Collection, which may exclude relevant publications indexed in other databases such as Scopus or PubMed. Because database coverage differs across Web of Science, Scopus, and PubMed, the findings should be interpreted as WoSCC-based trends rather than a complete representation of the global implant literature. Citation counts are also influenced by multiple external factors, including publication age, journal visibility, language, research trends, collaboration patterns, and self-citation, and therefore do not necessarily reflect methodological quality, clinical relevance, or scientific importance. In addition, document types in Web of Science are not necessarily mutually exclusive, which may affect the interpretation of document-type totals. Furthermore, the use of a fixed citation threshold (*TC*_2024_ ≥ 100) is a pragmatic but arbitrary criterion that may favor older and more visible publications, and no sensitivity analysis or field-normalized approach was applied. Citation-based indicators are influenced by time since publication and disciplinary citation practices, and no normalization by publication year, field, or document type was performed, limiting cross-comparison of citation impact. In addition, keyword-based retrieval strategies may not capture all relevant implant-related publications. Finally, network-based analyses, including co-authorship, co-citation, bibliographic coupling, and keyword co-occurrence mapping, were not performed. Consequently, the present study should be regarded as a descriptive bibliometric analysis rather than a network science or science-mapping study. Despite these limitations, the use of standardized bibliometric methods and reproducible search strategies strengthens the reliability of the findings. In addition, no inferential statistical analyses were conducted, and the results are therefore descriptive rather than explanatory.

This study has several strengths. First, it applies standardized and reproducible bibliometric indicators, including *TC_year_* and *Y*-index, which enhance methodological robustness and comparability across studies. Second, the use of a large dataset of highly cited publications provides a comprehensive overview of influential research within implant dentistry. Third, the application of the “front page” filter improves the specificity of the search strategy by reducing the inclusion of irrelevant records. Finally, the integration of bibliometric findings with clinically relevant themes enhances the translational value of the study for evidence-based practice.

## 5. Conclusions

This bibliometric analysis indicates that highly cited dental implant research is characterized by strong representation of biological, material, and clinical domains. Influential contributions reflect the integration of biological mechanisms, material science, and long-term clinical outcomes, highlighting the interdisciplinary and translational nature of implant dentistry. The concentration of highly cited research within specific countries, institutions, and author groups underscores the role of established centers of expertise in shaping the field. Overall, the findings provide insight into influential contributors, publication patterns, and prominent research themes, and may help identify areas of sustained scientific visibility and inform future research directions within implant dentistry.

## Figures and Tables

**Table 1:** Citations and authors according to the document type.

Document Type	*TP*	%	*AU*	*APP*	*TC*_2024_	*CPP*_2024_
Article	1016	77	5054	5.0	191,622	189
Review	302	23	1237	4.1	68,404	227
Proceedings paper	89	6.7	374	4.2	24,286	273
Editorial material	4	0.30	10	2.5	646	162
Note	2	0.15	7	3.5	399	200
Retracted publication	1	0.076	3	3.0	157	157

*TP*: total number of highly cited publications; %: percentage of articles in all highly cited articles; *AU*: total number of authors; *APP*: average number of authors per publication; *TC*_2024_: total number of citations from WoSCC since publication year until the end of 2024; *CPP*_2024_: average number of citations per publication (*TC*_2024_/*TP*); N/A: not available.

**Table 2:** The top ten most productive journals.

Journal	*TP* (%)	*IF*_2023_	*APP*	*CPP*_2024_	Web of Science Category
Clinical Oral Implants Research	278 (27)	4.8	4.9	182	Dentistry, oral surgery and medicine Biomedical engineering
International Journal of Oral & Maxillofacial Implants	135 (13)	1.7	4.0	179	Dentistry, oral surgery and medicine
Journal of Periodontology	103 (10)	4.2	5.2	186	Dentistry, oral surgery and medicine
Journal of Clinical Periodontology	64 (6.3)	5.8	5.7	252	Dentistry, oral surgery and medicine
Biomaterials	46 (4.5)	12.8	6.2	221	Biomedical engineering Biomaterials materials science
Clinical Implant Dentistry and Related Research	39 (3.8)	3.7	4.8	157	Dentistry, oral surgery and medicine
Journal of Dental Research	29 (2.9)	5.7	5.3	222	Dentistry, oral surgery and medicine
Journal of Oral and Maxillofacial Surgery	29 (2.9)	2.3	4.1	182	Dentistry, oral surgery and medicine
Journal of Prosthetic Dentistry	21 (2.1)	4.3	3.6	166	Dentistry, oral surgery and medicine
Acta Biomaterialia	20 (2.0)	9.4	6.4	202	Biomedical engineering Biomaterials materials science

*TP*: total number of highly cited articles; %: percentage of articles in all articles; *IF*_2023_: journal impact factor in 2023; *APP*: average number of authors per publication; *CPP*_2024_: average number per publication (*TC*_2024_/*TP*).

**Table 3:** Top 10 most productive countries.

Country	*TP*	*TP* (n = 1011)		*IP*_C_ (n = 694)		*CP*_C_ (n = 317)		*FP* (n = 1011)		*RP* (n = 1004)		*SP* (n = 25)	
		*R* (%)	*CPP*_2024_	*R* (%)	*CPP*_2024_	*R* (%)	*CPP*_2024_	*R* (%)	*CPP*_2024_	*R* (%)	*CPP*_2024_	*R* (%)	*CPP*_2024_
USA	308	1 (30)	208	1 (22)	197	1 (49)	219	1 (22)	204	1 (22)	203	1 (40)	173
Switzerland	196	2 (19)	225	3 (10)	208	2 (39)	235	2 (12)	227	2 (12)	226	4 (8)	137
Sweden	147	3 (15)	202	2 (11)	193	3 (23)	211	4 (9.5)	197	4 (10)	199	2 (16)	149
Italy	129	4 (13)	172	4 (10)	173	5 (18)	171	3 (10)	170	3 (11)	169	7 (4.0)	123
Germany	125	5 (12)	200	5 (8.2)	159	4 (21)	234	5 (8.1)	171	5 (8.2)	169	N/A	N/A
Spain	57	6 (5.6)	213	11 (2.7)	186	6 (12)	227	8 (3.3)	170	8 (3.1)	174	N/A	N/A
Netherlands	57	6 (5.6)	183	6 (4.5)	158	10 (8.2)	214	6 (4.0)	157	6 (4.1)	157	N/A	N/A
China	56	8 (5.5)	200	9 (3.0)	184	7 (11)	209	10 (2.8)	175	10 (2.9)	177	N/A	N/A
Belgium	51	9 (5.0)	201	8 (3.5)	171	9 (8.5)	228	7 (3.6)	181	7 (3.6)	176	N/A	N/A
UK	50	10 (4.9)	202	10 (2.9)	210	8 (9.5)	196	11 (2.6)	198	11 (2.7)	197	3 (12)	307

*TP*: number of total highly cited articles; *TP R* (%): total number of articles and the percentage of total articles; *IP*_C_ *R* (%): rank and percentage of single-country articles in all single-country articles; *CP*_C_ *R* (%): rank and percentage of internationally collaborative articles in all internationally collaborative articles; *FP R* (%): rank and the percentage of first-author articles in all first-author articles; *RP R* (%): rank and the percentage of corresponding-author articles in all corresponding-author articles; *SP R* (%): rank and the percentage of single-author articles in all single-author articles; *CPP*_2024_: average number of citations per publication (*CPP*_2024_ = *TC*_2024_/*TP*); N/A: not available.

**Table 4:** Top 10 most productive institutions.

Institution	*TP*	*TP* (n = 641)		*IP*_I_ (n = 210)		*CP*_I_ (n = 431)		*FP* (n = 641)		*RP* (n = 609)		*SP* (n = 32)	
		*R* (%)	*CPP*_2024_	*R* (%)	*CPP*_2024_	*R* (%)	*CPP*_2024_	*R* (%)	*CPP*_2024_	*R* (%)	*CPP*_2024_	*R* (%)	*CPP*_2024_
Univ Bern	126	1 (12)	249	1 (7.3)	244	1 (16)	250	1 (6.6)	226	1 (6.4)	227	3 (4.0)	117
U Gothenburg	71	2 (7.0)	237	2 (5.8)	189	2 (7.8)	259	2 (4.1)	226	2 (4.1)	228	N/A	N/A
Univ Zurich	51	3 (5.0)	236	5 (2.4)	169	3 (6.7)	250	3 (2.6)	229	3 (2.7)	225	N/A	N/A
Univ Milan	37	4 (3.7)	192	3 (4.5)	208	8 (3.2)	178	4 (2.4)	212	4 (2.0)	218	3 (4.0)	123
Univ Texas	35	5 (3.5)	230	20 (0.79)	110	4 (5.1)	241	5 (1.8)	202	6 (1.5)	219	3 (4.0)	104
U Michigan	34	6 (3.4)	263	4 (2.6)	158	6 (3.8)	307	6 (1.6)	177	5 (1.6)	177	N/A	N/A
Harvard Univ	31	7 (3.1)	177	8 (1.6)	147	5 (4.0)	184	8 (1.4)	151	9 (1.3)	152	N/A	N/A
UCLA	27	8 (2.7)	243	7 (2.1)	186	9 (3.0)	267	8 (1.4)	224	8 (1.4)	216	N/A	N/A
Gothenburg U	27	8 (2.7)	167	5 (2.4)	194	10 (2.9)	154	7 (1.5)	185	6 (1.5)	185	3 (4.0)	117
U Hong Kong	24	10 (2.4)	232	64 (0.26)	138	7 (3.7)	236	58 (0.30)	132	54 (0.30)	132	N/A	N/A

*TP*: total number of articles; *TP R* (%): total number of articles and percentage of total articles; *IP*_I_ *R* (%): rank and percentage of single-institution articles in all single-institution articles; *CP*_I_ *R* (%): rank and percentage of inter-institutionally collaborative articles in all inter-institutionally collaborative articles; *FP R* (%): rank and percentage of first-author articles in all first-author articles; *RP R* (%): rank and percentage of corresponding-author articles in all corresponding-author articles; *SP R* (%): rank and the percentage of single-author articles in all single-author articles; *CPP*_2024_: average number of citations per publication (*CPP*_2024_ = *TC*_2024_/*TP*); N/A: not available. Univ Bern: University of Bern, Switzerland; U Gothenburg: University of Gothenburg, Sweden; Univ Zurich: University of Zurich, Switzerland; Univ Milan: University of Milan, Italy; Univ Texas: University of Texas, USA; U Michigan: University of Michigan, USA; Harvard Univ: Harvard University, USA; UCLA: University of California, Los Angeles, USA; Gothenburg U: Gothenburg University, Sweden; U Hong Kong: University of Hong Kong, China.

**Table 5:** Top 20 productive authors with at least 14 highly cited articles.

Author	*TP* (n = 1016 Articles)		*FP* (n = 1016 Articles)		*RP* (n = 711 Articles)		*h* (n = 711)	Rank (*j*)
	Rank (*TP*)	*CPP*_2024_	Rank (*FP*)	*CPP*_2024_	Rank (*RP*)	*CPP*_2024_		
N.P. Lang	1 (46)	235	60 (2)	181	22 (3)	132	1.249	41 (4)
D. Buser	2 (44)	265	1 (9)	502	5 (8)	201	1.107	5 (12)
T. Berglundh	3 (39)	274	1 (9)	398	1 (15)	251	1.081	1 (23)
C.H.F. Hämmerle	4 (28)	223	16 (4)	189	48 (2)	145	π/4	41 (4)
R.E. Jung	5 (23)	235	1 (9)	316	4 (9)	316	π/4	2 (18)
H.L. Wang	6 (22)	264	147 (1)	105	8 (6)	158	1.406	16 (7)
D.L. Cochran	6 (22)	256	10 (6)	230	10 (5)	129	1.030	14 (8)
U. Brägger	6 (22)	212	30 (3)	209	108 (1)	160	π/2	379 (1)
A. Piattelli	9 (21)	163	16 (4)	145	2 (10)	159	1.279	4 (13)
G.E. Salvi	9 (21)	271	16 (4)	188	48 (2)	197	π/4	41 (4)
H.P. Weber	11 (19)	180	30 (3)	160	108 (1)	112	π/4	108 (2)
M. Quirynen	11 (19)	225	30 (3)	147	10 (5)	156	1.190	16 (7)
J. Lindhe	11 (19)	174	N/A	N/A	N/A	N/A	0	699 (0)
F. Schwarz	14 (16)	295	8 (7)	273	8 (6)	290	π/4	5 (12)
D. Van Steenberghe	15 (15)	182	30 (3)	255	108 (1)	303	π/2	379 (1)
M.M. Bornstein	15 (15)	185	1 (9)	143	19 (4)	163	0.5191	8 (11)
A. Scarano	17 (14)	185	147 (1)	495	N/A	N/A	0	699 (0)
L. Sennerby	17 (14)	193	147 (1)	270	N/A	N/A	0	699 (0)
M. Chiapasco	17 (14)	206	8 (7)	242	22 (3)	283	π/4	22 (6)
I. Abrahamsson	17 (14)	156	60 (2)	159	48 (2)	159	π/4	41 (4)

*TP*: total number of highly cited articles; *FP*: first-author highly cited articles; *RP*: corresponding-author highly cited articles; *CPP*_2024_: average number of citations per publication (*CPP*_2024_ = *TC*_2024_/*TP*); *j*: a *Y*-index constant related to the publication potential; *h*: a *Y*-index constant related to the publication characteristics; N/A: not available.

**Table 6:** Top 20 most frequently used words in highly cited article titles and author keywords.

Words in Title	*TP*	*R* (%) n = 1016	Author Keywords	*TP*	*R* (%) n = 885
Bone	247	1 (24)	Osseointegration	94	1 (11)
Titanium	157	2 (15)	Peri-implantitis	94	1 (11)
Clinical	148	3 (15)	Titanium	86	3 (10)
Prospective	91	4 (9)	Immediate loading	36	4 (4.1)
Surface	76	5 (7.5)	Bone regeneration	30	5 (3.4)
Evaluation	67	6 (6.6)	Periodontitis	23	6 (2.6)
Peri-implant	67	6 (6.6)	Systematic review	21	7 (2.4)
Immediate	66	8 (6.5)	Bone	19	8 (2.1)
Sinus	61	9 (6.0)	Clinical trial	19	8 (2.1)
Augmentation	52	10 (5.1)	Survival	19	8 (2.1)
Patients	52	10 (5.1)	Histology	18	11 (2.0)
Review	52	10 (5.1)	Accuracy	17	12 (1.9)
Maxillary	50	13 (4.9)	Bone loss	17	12 (1.9)
Treatment	50	13 (4.9)	Complications	17	12 (1.9)
Peri-implantitis	49	15 (4.8)	Osteoblasts	17	12 (1.9)
Placement	49	15 (4.8)	Sinus floor elevation	17	12 (1.9)
Tissue	49	15 (4.8)	Biological complications	16	17 (1.8)
Edentulous	48	18 (4.7)	Guided bone regeneration	16	17 (1.8)
Trial	47	19 (4.6)	Hydroxyapatite	16	17 (1.8)
Influence	44	20 (4.3)	Implant stability	16	17 (1.8)

*TP*: number of articles; %: percentage; *R*: rank.

## Data Availability

No new data were created or analyzed in this study. Data sharing is not applicable to this article.

## Supplementary Materials

The following supporting information can be downloaded at: https://www.mdpi.com/article/10.3390/healthcare14101301/s1, Table S1: The BIBLIO-checklist, which ensures the highest standards of design, conduct, and reporting of bibliometric studies. Refs. [21,55,56,57,58,59,60,61,62] are cited in the supplementary material file.

## Abbreviations

The following abbreviations are used in this manuscript:

APP	authors per publication
C	number of citations
CP	number of internationally or inter-institutionally collaborative articles
CPP	average number of citations per publication
JCR	Journal Citation Reports
FP	number of first-author articles
IF	Journal impact factors
IP	number of articles published by a single country or institution
RP	number of corresponding-author articles
SCI-EXPANDED	Science Citation Index Expanded
SP	number of single-author articles
TC	total citations
TP	total number of publications
TS	topic field
UK	United Kingdom
WoSCC	Web of Science Core Collection
